# Natural Products with Activity against Lung Cancer: A Review Focusing on the Tumor Microenvironment

**DOI:** 10.3390/ijms221910827

**Published:** 2021-10-07

**Authors:** Yue Yang, Ning Li, Tian-Ming Wang, Lei Di

**Affiliations:** Inflammation and Immune Mediated Diseases Laboratory of Anhui Province, School of Pharmacy, Anhui Medical University, Hefei 230032, China; yangyue9288@163.com (Y.Y.); wtm1818@163.com (T.-M.W.)

**Keywords:** natural products, tumor microenvironment (TME), lung cancer, phytochemicals, botanical agents

## Abstract

Lung cancer is one of the most prevalent malignancies worldwide. Despite the undeniable progress in lung cancer research made over the past decade, it is still the leading cause of cancer-related deaths and continues to challenge scientists and researchers engaged in searching for therapeutics and drugs. The tumor microenvironment (TME) is recognized as one of the major hallmarks of epithelial cancers, including the majority of lung cancers, and is associated with tumorigenesis, progression, invasion, and metastasis. Targeting of the TME has received increasing attention in recent years. Natural products have historically made substantial contributions to pharmacotherapy, especially for cancer. In this review, we emphasize the role of the TME and summarize the experimental proof demonstrating the antitumor effects and underlying mechanisms of natural products that target the TME. We also review the effects of natural products used in combination with anticancer agents. Moreover, we highlight nanotechnology and other materials used to enhance the effects of natural products. Overall, our hope is that this review of these natural products will encourage more thoughts and ideas on therapeutic development to benefit lung cancer patients.

## 1. Introduction

Lung cancer, which is classified into non-small cell lung cancer (NSCLC) and small cell lung cancer (SCLC), is one of the most prevalent malignancies worldwide in terms of both incidence and mortality (18.0% of total cancer deaths) [1]. Therapeutic options for this cancer are surgery, radiation therapy, and systemic treatments including chemotherapy, targeted therapy, hormonal therapy, and immunotherapy. Because the diagnosis of SCLC is rarely localized, surgical resection plays a small role in its treatment. Most patients with SCLC receive chemotherapy. Approximately 56% of patients with stage I and II NSCLC undergo surgery. For patients with stage III NSCLC, only 18% are treated with surgery, while most (62%) undergo chemotherapy or radiotherapy. Immune and targeted therapeutic drugs are used for the treatment of advanced NSCLC, but some drugs are only used for the treatment of cancers with specific gene mutations [2]. Despite the tremendous efforts in research on the treatment of lung cancer, the incidence and mortality rates of lung cancer have not decreased significantly [3]. Therefore, it is necessary to find more treatment strategies and drugs for lung cancer.

**Figure 1:**
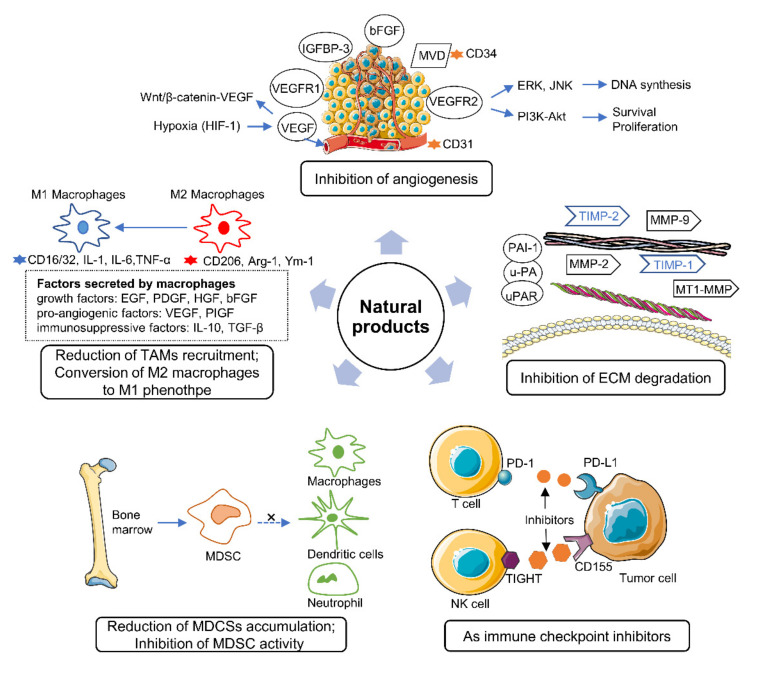
Modulation of the TME by natural products.

The tumor microenvironment (TME) is a complex ecosystem consisting of the vasculature, extracellular matrix (ECM), cytokines and growth factors, and many different populations of stromal cells, such as myeloid-derived suppressor cells (MDSCs), tumor-associated macrophages (TAMs), and tumor-associated fibroblasts (TAFs) [4]. Over the past decade, the TME has been recognized as playing key roles in lung cancer initiation and progression [5,6]. As the composition of the TME is heterogeneous, interactions between cancer and stromal cells in the microenvironment regulate the main characteristics of cancer, including its immune suppression, angiogenesis, and inflammation properties [7]. These properties support the growth, invasion, and metastasis of cancer cells. Therefore, the role of the TME in lung cancer has received increasing attention. The TME is regarded as a target-rich environment in the development of new anticancer drugs. Strategies that target cancer cells are considered as treatment avenues. Moreover, different from cancer cells, the stromal cells in the TME are genetically stable, therefore, they are attractive therapeutic targets that are associated with reductions in drug resistance and tumor recurrence [8]. More and more studies are focusing on the TME as a target for drug research and development (Figure 1).

Natural products are precious gifts from nature to mankind. They include extracts of animals and plants, metabolites of insects, marine organisms, and microorganisms, as well as many chemical components found endogenously in humans and animals. In addition, traditional Chinese medicine (TCM) is based on the combination of natural products and TCM theory. Natural products have always been an important source of drug discovery. According to the latest statistics on drugs approved by the Food and Drug Administration (FDA) in the United States, many prescription medicines used for treatment originate from natural products. From 1946 to 2019, more than 50% of newly approved drugs were natural small molecules [9]. Plant preparations and Chinese medicines are multi-component, multi-channel, and multi-target products. Due to their diverse structures and activities, natural products continue to attract researchers’ attention. Although the TME has been widely studied, the natural products that target and regulate the TME of lung cancer have not been systematically summarized. In this review, we describe the antitumor effect of natural products on the TME in lung cancer. We summarize relevant natural products, including descriptions of their anti-tumor actions in terms of modulating the TME in lung cancer when given alone (Table 1), in combination with anticancer drugs (Table 2), and in combination with materials such as nanomaterials.

## 2. Effects of Natural Products on the Tumor Microenvironment

### 2.1. Natural Products Involved in Angiogenesis Inhibition

Angiogenesis refers to the formation of new blood vessels from the pre-existing vasculature, and is an important hallmark in the development of malignant tumors [83]. Tumor growth and subsequent metastasis require nutrients and oxygen supplied by an elaborate network of blood vessels [84]. Endothelial cells are the main cells that are directly involved in angiogenesis. Cytokines in the TME, such as vascular endothelial growth factor (VEGF), can induce angiogenesis through different mechanisms. Angiogenesis inhibitors targeting various components of the VEGF pathway have been developed. These inhibitors were found and designed to inhibit VEGF ligand, VEGFR (vascular endothelial growth factor receptor), and downstream signal elements. VEGFR1 and VEGFR2 are VEGF receptors, of which VEGFR2 (KDR/Flk) has stronger tyrosine kinase activity [85]. VEGFR2 is the major signal transducer in angiogenesis, and its actions include the activation of ERK and JNK for DNA synthesis and the activation of PI3K-Akt for survival and proliferation. The VEGF antibody bevacizumab, the VEGFR2 antibody ramucirumab, and the tyrosine kinase inhibitor nintedanib have been approved for clinical use [5]. However, in some cases, it is difficult for these compounds to penetrate into the smallest blood vessels of tumor tissue. Due to the great difference in penetration of angiogenesis inhibitors in different tumor tissues, the drug concentration can only reach an effective concentration in some tumors. Therefore, the clinical benefit of such compounds is limited [86]. Researchers are continuously searching for natural antiangiogenic agents. It is hoped that natural products could block the formation of new blood vessels to prevent or slow down the growth or metastasis of cancer.

**Figure 2:**
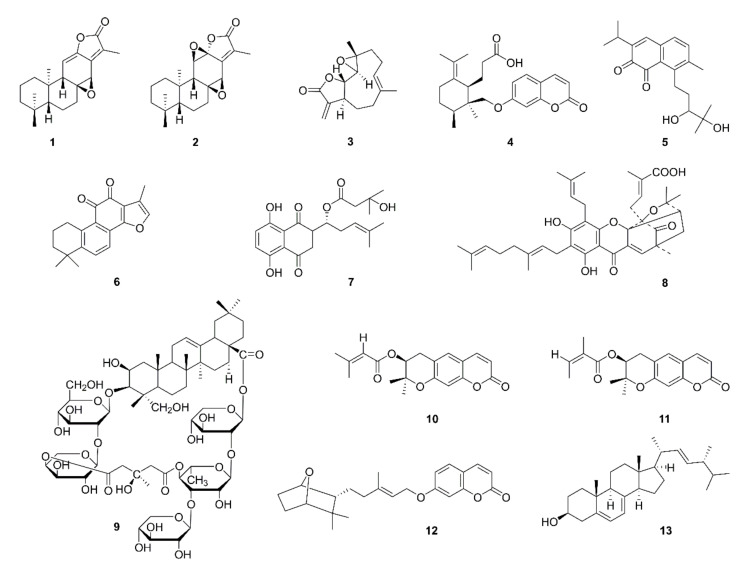
Chemical structures of natural compounds targeting angiogenesis (**1**–**13**).

Jolkinolide A (**1**) and jolkinolide B (**2**), diterpenoids isolated from the roots of *Euphorbia fischeriana*, were shown to decrease the expression of VEGF and inhibit the Akt/STAT3/mTOR signaling pathway in A549 cells. The inhibitory effect of jolkinolide B is more obvious than that of jolkinolide A. The medium of A549 cells stimulated with either jolkinolide A or jolkinolide B was found to inhibit the proliferation and migration of human umbilical vein endothelial cells (HUVECs) in a concentration dependent manner [10]. Parthenolide (**3**), a sesquiterpene lactone extracted from *Tanacetum parthenium*, was shown to inhibit the proliferation of A549 cells in the absence and presence of nicotine. The activity of capsase-3, a key enzyme and indicator in apoptosis induction pathways, was found to be enhanced with parthenolide treatment. Essential protein VEGF levels of angiogenesis were also significantly reduced [11]. The LLC mouse model was established by Kim et al. to show that galbanic acid (**4**) extracted from *Ferula assafoetida* inhibits angiogenesis and tumor growth and reduces microvessel density (MVD) index CD34 expression in vivo. Galbanic acid was shown to disrupt the VEGF-induced tube formation of HUVECs. The phosphorylation of downstream signaling compounds such as p38MAPK, JNK and Akt was found to be decreased by galbanic acid treatment in VEGF-treated HUVECs in vitro [12]. Salvicine (**5**) was shown to decrease mRNA expression of basic fibroblast growth factor (bFGF), an enhancer of angiogenesis, but it was not associated with a change in VEGF expression [13]. Natural compounds targeting VEGFR2 include tanshinone IIA (**6**) [14], β-hydroxyisovalerylshikonin (**7**) [15], isogambogenic acid (**8**) [16], tubeimoside-1 (**9**) [17], decursin (**10**) and decursinol angelate (**11**) [18]. Farnesiferol C (**12**) was shown to exert antitumor activity and antiangiogenic actions by targeting VEGFR1 [19]. Takaku et al. reported that ergosterol (**13**) or its metabolites might be involved in neovascularization inhibition using an LLC model [20]. The chemical structures of antiangiogenic compounds identified in recent research are displayed in Figure 2.

Plant preparations are a promising choice for the development of more effective chemoprevention and chemotherapy strategies. Grape seed proanthocyanidins (GSPs), a mixture of flavanols/polyphenols, mainly containing proanthocyanidins (89%), can be used as dietary supplements with antioxidant and anticancer properties [21,22]. Akhtar et al. showed that GSPs inhibit the proliferation of a variety of human NSCLC cells and mouse Lewis lung carcinoma (LLC) cells in a dose- and time-dependent manner in vitro. GSPs were not found to inhibit the proliferation of BEAS-2B normal human bronchial epithelial cells. GSPs were also shown to inhibit the tumor growth of A549 and H1299 xenografts in vivo. The results of a tumor tissue immunohistochemical assay showed a reduction in VEGF protein expression with GSPs treatment. CD31 contributes to the formation of neovascularization and is therefore a biomarker of angiogenesis [23]. Immunofluorescence staining showed that the expression trend of CD31 is consistent with that of VEGF after treatment with GSPs. This further verified that GSPs could inhibit angiogenesis. Moreover, the protein level of IGFBP-3 with antiangiogenic antitumor activity in lung tumor tissues and plasma increased with GSPs treatment [24,25]. Khan et al. studied the antitumor effect of pomegranate fruit extract on growth, progression, angiogenesis, and signaling pathways in a primary lung tumor mouse model. Pomegranate fruit extract was found to effectively inhibit the incidence of lung cancer and reduce the activation of PI3K/Akt, MAPK, NF-κB, mTOR signaling, and c-met. Additionally, the expression of markers of cell proliferation or angiogenesis, including Ki67, PCNA, VEGF, iNOS, and CD31, was also reduced. Thus, pomegranate fruit extract exerts tumor growth inhibition and angiogenesis effects through multiple signaling pathways [26]. Extracts of *Astragali Mongolici* and *Rhizoma Curcumae* were found to inhibit LLC growth and angiogenesis in a xenograft mouse model through the reduction of tumor MVD; decreased expression of VEGF; and activation inhibition of p38MAPK, ERK1/2, and JNK [27]. Hypoxia can promote angiogenesis by increasing the expression and secretion of VEGF [87]. Thus, hypoxia-inducible factor-1 (HIF-1) plays a key role in tumor angiogenesis. *Scutellaria barbata* extract was reported to inhibit angiogenesis by decreasing the expression of VEGF, HIF-1α, and the phosphorylated upstream signal mediator Akt in lung tumors [28]. *Ginkgo biloba* exocarp extracts were found to inhibit angiogenesis by blocking the Wnt/β-catenin-VEGF signaling pathway in LLC. mRNA expression levels of VEGF and VEGFR2 and protein expression levels of CD34, Wnt3a, and β-catenin were all decreased [29]. Green tea was shown to inhibit angiogenesis through reductions of MVD and VEGF in A/J mice [30].

The an-te-xiao capsule, which accounts for all alkaloids in *Solanum lyratum*, is used as an antineoplastic medicine in China. The an-te-xiao capsule was shown to prolong the survival time of Lewis tumor mice with no acute oral toxicity. In the presence or absence of VEGF, the migration, invasion, and tube formation of tumor-derived vascular endothelial cells (Td-ECs) were shown to be suppressed in A549, H460, and H520 cells when an an-te-xiao capsule was taken. Secretion of VEGF and phosphorylation of VEGFR2 were also inhibited [31]. Another Chinese medicine, the erbanxiao solution, was shown to significantly inhibit tumor angiogenesis in lung cancer patients, possibly by changing levels of VEGF, bFGF, and TNF-α [32]. Some other natural products that have shown growth inhibition or anti-angiogenesis effects by regulating the expression of VEGF and related signaling molecules include the Korean herbal cocktail ka-mi-kae-kyuk-tang [33] and Chinese medicines Qingzaojiufei decoction [34] and Yiqichutan formula [35].

### 2.2. Natural Products Control ECM Degradation

The ECM, consisting of collagen, glycosaminoglycans, proteoglycans, and laminin, is the primary component of the TME and is found in the interstitial and epithelial vessels. On the one hand, the ECM mediates interactions between cancer cells and stromal cells, promoting carcinogenesis. On the other hand, the ECM is an important barrier against tumor metastasis in tissues [88]. Degradation of the ECM promotes cancer cells to traverse the ECM and migrate into blood vessels. Then, under the activity of some cytokines, cancer cells pass through the vessel wall and extravasate to secondary sites where they continue to proliferate and form metastatic lesions [89]. Different types of proteases can cause the degradation of the ECM, the most important of which are the matrix metalloproteinases (MMPs). MMPs, a family of zinc-dependent endopeptidases produced by fibroblasts, epithelial cells, and immune cells, degrade various subtypes of collagen as well as other elements of the ECM. The urokinase-type plasminogen activator (u-PA), a key proteolytic enzyme, is known to involve the degration of ECM and convert proMMPs to active MMPs including MMP-2, which is a member of the MMP family. Membrane type 1–matrix metalloproteinase (MT1-MMP), the expression of which is abnormal in tumors, is involved in the regulation of MMP-2 activity [90]. MMP-9, another member of the MMP family, has certain value as a biomarker of various cancers [91]. Thus, inhibiting the activity or expression of MMPs may help to suppress tumor invasion and metastasis.

**Figure 3:**
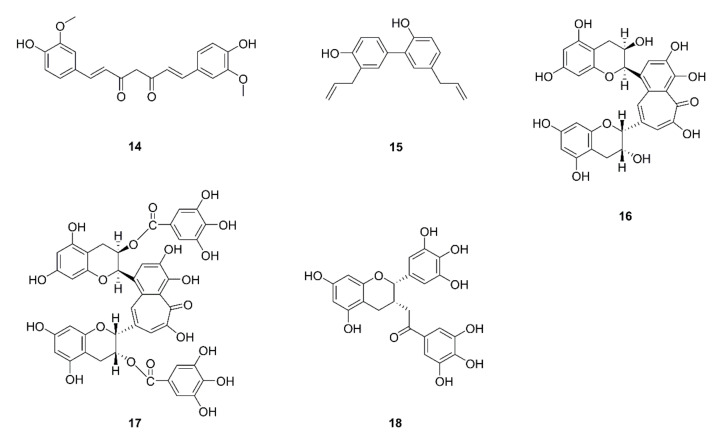
Chemical structures of natural compounds targeting the ECM (**14**–**18**).

In many cancers, including lung cancer, GLUT1 (glucose transporter 1) is overexpressed and regarded as a prognostic indicator. Curcumin (**14**) is a widely studied natural product with diverse activities [92]. Liao et al. reported that protein and mRNA expression of GLUT1, MT1-MMP, and MMP2 reduced in A549 cells following curcumin treatment at concentrations of 15 and 30 μM. Moreover, the anti-migration and anti-invasion effects of curcumin were shown to be damaged and MT1-MMP and MMP2 expression was up-regulated in GLUT1-overexpressed A549 cells. Consistent with the in vitro results, following curcumin treatment, the metastatic rate in nude mice that were untreated and transfected with empty vector A549 cells was shown to be about 50%, while the metastatic rate was 84% in nude mice bearing A549 cells and transfected with pcDNA3.1-GLUT1. The results showed that the overexpression of GLUT1 hinders the anti-metastasis effect of curcumin. Curcumin was shown to suppress migration and invasion by modulating the GLUT1/MT1-MMP/MMP2 pathway in A549 cells [36]. Another active compound, honokiol (**15**), derived from *Magnolia officinalis*, was found to inhibit migration and invasion by disrupting the expression of MMP-9 and Hsp90/MMP-9 interactions mediated by HDAC6 in H1299 cells. Honokiol was shown to promote ubiquitin–proteasome degradation of MMP-9 rather than inhibiting its transcription process. HDAC6 is a special deacetylase that regulates protein stability of Hsp90. Its actions are associated with the activation of MMP-2/9 through protein–protein interactions. Honokiol was shown to inhibit the expression of acetyl-α-tubulin, which is a specific substrate of HDAC6. Using a cell model, it was further proven that MMP-9 is regulated by HDAC6 [37]. In some earlier studies about natural active components, theaflavin (**16**) and theaflavin digallate (**17**), which are biologically active derivatives from black tea, were found to exert anti-metastasis effects through inhibiting type IV collagenase in LL2-Lu3 mouse LLC cells [38]. The green tea polyphenol (-)-Epigallocatechin-3-gallate (EGCG, **18**), which has a variety of activities, has been widely studied [39]. Deng et al. reported that EGCG exerts an anti-invasion effect by inhibiting mRNA and protein levels of MMP-2 via the JNK pathway in CL1-5 cells, which are highly invasive. EGCG was shown to suppress the activity of the MMP-2 promoter in a dose-dependent manner. Moreover, EGCG was found to enhance the anticancer effects of docetaxel and reduce MMP-2 expression [40]. Shi et al. reported that EGCG suppresses migration and invasion through inhibition of the epithelial-mesenchymal transition (EMT) and angiogenesis induced by nicotine [41]. The chemical structures of compounds targeting the ECM are displayed in Figure 3.

Steroidal saponins extracted from *Paris polyphylla* (PPSS) were shown to inhibit A549 cell growth, adhesion, migration and invasion in a concentration-dependent manner. The anti-invasive mechanism underlying these processes is that PPSS reduces the protein expression and activity of MMP-2 and MMP-9 [42]. Methanolic extract of *Euchelus asper* was also shown to exert anti-proliferative activities by decreasing the expression of MMP-2 and MMP-9 in vitro [43]. Another study reported that *Phyllanthus urinaria* extracts (PUE) suppress the migration and invasion of A549 and LLC cells through reduced expression of MMP-2, MMP-9, and u-PA [44]. Rose is one of the most important ornamental plants. A previous study reported that *Rosa gallica* petal extract (RPE) inhibits the proliferation, metastasis, and invasion of A375 cells by reducing the expression and activity of MMP-2 and -9. Different from the PUE, RPE was also shown to modulate the EGFR-MAPK and mTOR-Akt signaling pathways [45]. *Viola Yedoensis* extract (VYE) was not only found to inhibit the activity of MMP-2, -9, and u-PA, it was also shown to suppress the protein expression levels of TIMP-2, TIMP-1, and PAI-1 in A549 and LLC cells. Further research showed that VYE inhibits the binding of NF-κB to DNA. Thus, the inhibition of NF-κB suppresses MMP-2 and u-PA expression and lung cancer cell invasion [46]. Focal adhesion kinase (FAK) was found to be overexpressed and activated in some late-stage cancers. Activated p-FAK has been shown to promote migration and invasion and modulate u-PA and MMPs [93]. Active ERK1/2 was also shown to promote MMP production [94]. Wu et al. reported that *Cinnamomum cassia*, a traditional food and medicinal plant, exhibits anti-metastasis ability through reduced phosphorylation of FAK and ERK1/2 as well as downregulating MMP-2 and u-PA in A549 and H1299 cells [47]. Chen et al. reported that *Duchesnea indica* extracts (DIE) inhibit the expression of p-ERK1/2 and p-FAK in A549 and H1299 cells, subsequently reducing the expression of u-PA and MMP-2 mediated by p-ERK1/2 and the expression of p-paxillin, vimentin, fibronectin, and N-cadherin. Additionally, the expression of the epithelial marker E-cadherin was found to increase. These changes in signal molecules by DIE were found to inhibit cell adhesion, migration, invasion and the epithelial–mesenchymal transition (EMT). In an A549-bearing nude mouse xenograft, tumor growth was shown to be efficiently retarded by DIE treatment compared with a control group. A higher level of E-cadherin and lower levels of MMP-2 and N-cadherin were examined in tumor tissues in DIE-treated mice [48]. A number of studies have shown that various botanical agents, such as fructus phyllanthi tannin fraction and butanol fraction extract of *Psidium cattleianum* leaf, influence the ECM by downregulating the expression and activity of MMP-2 and -9 as well as the activation of ERK1/2. Fructus phyllanthi, the dried ripened fruit of *Phyllanthus emblica*, which has been used for thousands of years in the Tibetan area, was shown to modulate the MAPK pathway by dose-dependently upregulating the expression of p-JNK in H1703 cells [49]. The butanol fraction extract of *Psidium cattleianum* leaves was shown to suppress the urokinase plasminogen activator receptor (uPAR) and MAPK signaling pathway [50]. *Terminalia catappa* leaf extract was found to inhibit the activity of MMP-2, -9 and u-PA and up-regulate the expression of the proteins TIMP-2 and PAI-1 [51]. The main ingredients in *Paris polyphylla* are steroid saponins known as *Rhizoma Paridis* saponins (RPS). An experiment in which T739 mice were injected subcutaneously with LA795 mouse lung adenocarcinoma cells showed that after RPS treatment, mRNA levels of MMP-2 and -9 were reduced and TIMP-2 was upregulated in tumor tissues [52]. *Selaginella tamariscina* extracts were shown to not only downregulate the activity of MMP2/9 and u-PA but also decrease the protein levels of TIMP and PAI in A549 and LLC cells [53]. There is evidence showing that metastasis can also be inhibited by regulating antioxidant enzymes [95]. *Ocimum sanctum* is generally known as “Holy basil” and is used in Ayurvedic medicine in India [96]. Ethanol extract of *Ocimum sanctum* (EEOS) has been shown to play roles in adhesion and invasion in LLC cells by inhibiting MMP-9 rather than MMP-2 activation. Meanwhile, antioxidant enzyme activity, including the activity of including SOD, CAT, and GSH-Px, was found to decrease in lung cancer tissues in LLC bearing mice treated with EEOS. Additionally, the ratio of GSH/GSSG was shown to decrease [54]. A unique experiment using an ex vivo approach demonstrated the suppression of metastasis and investigated the mechanisms underlying the actions of serum metabolites from rhubarb, the dried root and rhizome of *Rheum palmatum*. First, serum metabolites were extracted from rats that had been administered rhubarb by gavage. Then, A549 cells were cocultured with rhubarb serum metabolites. The results of a wound healing assay, zymography, RT-PCR, and Western blot analysis showed that rhubarb serum metabolites suppress the activity and expression of MMP-2 and u-PA. Protein expression levels of phosphorylated NF-κB and c-Jun were reduced. Many studies have shown that transcription factors such as NF-κB, c-Jun, and AP-1 are involved in the transcriptional regulation of MMP-2 and -9 [97,98,99,100]. It has been indicated that some active components of rhubarb serum metabolism inhibit the activity of MMP-2 by inhibiting the u-PA and NF-κB/c-Jun pathway. These components were shown to block motility and inhibit the metastasis of A549 cells in vitro. Using a lung metastatic mouse model, the number of metastatic nodules in the lungs of rhubarb-treated mice was shown to be reduced compared with a control group [55].

Fuzheng Kang-Ai decoction (FZKA), a classic Chinese herbal medicine, is used to treat cancers. Li et al. reported that FZKA inhibits the metastasis of H1650, A549, and PC-9 cells through inhibition of the STAT3/MMP-9 pathway and EMT. After FZKA treatment, the activity and expression of MMP-9 were shown to be reduced. Additionally, activation of signal transducer and activator of transcription 3 (STAT3) was inhibited. However, the overexpression of STAT3 was demonstrated to rescue the activity of MMP-9. On the other hand, the expression of the mesenchymal markers N-cadherin and vimentin was found to reduce following FZKA treatment [56]. Another Chinese herbal formula, Yifei Tongluo (YFTL), was found to inhibit tumor growth, metastasis, and angiogenesis; prolong survival; and improve immunity through multiple signaling pathways in Lewis lung cancer bearing mice. The expression of the major angiogenesis-associated protein VEGF was found to be inhibited in both tumor tissues and serum. YFTL was shown to induce significant reductions in MMP-2, MMP-9, N-cadherin, and vimentin expression levels as well as increasing E-cadherin expression. CD4^+^ and CD8^+^ T lymphocytes are the major components of T cell-mediated anti-tumor immunity [101]. Natural killer (NK) cells also play a role in antitumor immunity [102]. CD4^+^, CD8^+^, and NK cells were found to increase following treatment with YFTL. The cytokine levels of IL-2, IFN-γ, IL-10, and TGF-β1, components that promote antitumor immunity through proinflammatory actions, were found to increase in the serum of tumor-bearing mice following treatment with YFTL. In addition, experimental results showed that YFTL inhibited the ERK1/2, TGF1/Smad2, and PI3K/Akt, pathways and upregulated the p38 and JNK pathways. Taken together, these YFTL-regulated factors have been shown to suppress tumor growth and metastasis in Lewis-tumor-bearing mice [57].

### 2.3. Natural Products Reduce the Accumulation of MDSCs

MDSCs, as regulators of the immune system, are a heterogeneous population of immature myeloid progenitors and precursors of dendritic cells, granulocytes, and macrophages [103,104]. Uncontrolled expansion of MDSCs disrupts the normal homeostasis of the immune system and eventually lead to tumor progression and the development of other diseases. In cancer patients, an increase in MDSCs has been shown to induce intense immunosuppressive activity through inhibiting the functions of NK cells and cytotoxic T lymphocytes (CTLs) [105,106]. MDSCs are classified into two subsets: monocytic-MDSC (M-MDSC) and granulocytic-MDSC (G-MDSC). Anti-MDSC treatments, such as abrogating suppressive activity or reducing the number of MDSCs in the tumor microenvironment, have been successfully used as anticancer therapy [107].

Resveratrol (**19**) is a polyphenol derived from grape skin and seeds that provides an abundance of health benefits [108]. Zhao et al. reported that resveratrol reduces G-MCSD accumulation by triggering its apoptosis and promotes CD8^+^IFN-γ^+^ cell expansion in Lewis lung carcinoma bearing mice. Resveratrol was also shown to impair the activity of CD8^+^ T cells, which are suppressed by MDSCs. Moreover, resveratrol was shown to enhance the differentiation of MDSCs separated from mice into CD11c^+^ (pro-inflammatory macrophage- or dendritic cell-like) and F4/80^+^ (macrophage marker) cells. Taken together, these results show that resveratrol inhibits the development of tumors in mice with Lewis lung carcinoma [58]. In another study of the same mouse model, Wu et al. provided evidence that silymarin (**20**) reduces the proportion of MDSCs in tumor tissues. Additionally, the mRNA expression levels of iNOS2, Arg-1 and MMP-9 were found to be reduced in tumor tissues, indicating that the function of MDSCs was suppressed. Mature T lymphocytes are mainly divided into CD4^+^ and CD8^+^ cells. CD8^+^ T cells are activated by CD4^+^ cells and migrate to the tumor site, exerting a cytotoxic effect. The study showed that silymarin increased the expression of CD8+ T cells in the tumor tissues compared to the control group [59]. Vitamin D (**21**) is a fat-soluble vitamin that regulates calcium, phosphate, and magnesium homeostasis, and influences elements of human health, including the immune response and tumorigenesis [109]. A study on COVID-19 patients showed that the absence of vitamin D increased the severity of the acute respiratory distress syndrome (ARDS) induced by cytokine storm. Vitamin D supplementation could affect the inflammatory responses of macrophages and MDSCs, inhibiting a strong inflammatory response and reducing the ARDS of COVID-19 patients [60].

**Figure 4:**
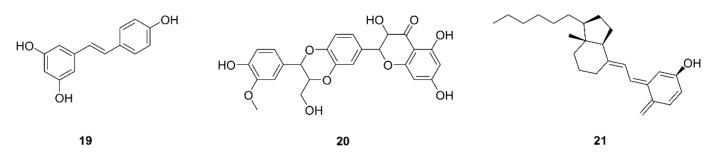
Chemical structures of natural compounds targeting MDSCs (**19**–**21**).

Polysaccharides are the main components of herbs. Polysaccharides extracted from herbal medicine have important medicinal value with their actions including immunity improvement and anti-tumor effects [110]. A homogeneous polysaccharide from *Ganoderma lucidum* (GLP) was reported to induce differentiation and reduce the accumulation of MDSCs through the CARD9-NF-κB-IDO signaling pathway, preventing lung cancer development in an LLC mouse model [61,111]. Polysaccharides come not only from plants but also from bacteria. Curdlan, composed of linear b-(1,3)-glycosidic linkages, is a bacterial polysaccharide produced by *Alcaligenes faecalis*. Rui et al. reported that curdlan can promote the differentiation of MDSCs into a more mature state. A reduction of MDSCs was found to downregulate immunosuppression in LLC-bearing mice, thus having an antitumor effect [62]. Besides polysaccharides, Ishiguro et al. reported that water extract from *Euglena gracilis* induced G-MDSC apoptosis and the differentiation of M-MDSCs into macrophages. Thus, the water extract stimulated host antitumor immunity and inhibited lung tumor growth [112]. Chemical structures of compounds targeting MDSCs are displayed in Figure 4.

The Ze-Qi-Tang formula (ZQT) has been used traditionally to treat respiratory diseases. Xu et al. firstly illustrated the immunomodulatory effect of ZQT in NSCLC. ZQT was found to induce G-MDSC apoptosis through the STAT3/S100A9/Bcl-2/caspase-3 signaling pathway, and it significantly reduced the number of MDSCs. ZQT was shown to remodel the immune tumor microenvironment by eliminating MDSCs and enriching T cells, prolonging survival and inhibiting tumor growth in a orthotopic mouse model of lung cancer [63].

### 2.4. Natural Products Regulate TAMs

Macrophages acquire diverse phenotypes in response to different stimuli generated by activated stromal cells or cancer cells in the microenvironment [113]. M1 macrophages promote antitumor responses, while M2 macrophages drive tumor progression. In the tumor microenvironment, TAMs are generally M2-polarized [114]. TAMs are one of the major cell populations within the stroma of various cancers. It was reported that having a high TAMs number is closely related to the presence of advanced cancer [115]. TAMs stimulate cell proliferation and promote angiogenesis and tumor metastasis by secreting various cytokines. For example, the proliferation of tumor cells is promoted by growth factors such as EGF, PDGF, HGF, and bFGF, which are secreted by macrophages. TAMs promote angiogenesis through the release of pro-angiogenic factors such as VEGF and PIGF. Tumor invasion is promoted by substrates and tumor aggregation factors, such as EGF. Immunosuppression is achieved by immunosuppressive factors, such as IL-10 and TGF-β [116]. Moreover, TAMs produce a series of proteases including u-PA and MMPs to degrade ECM and promote cancer cell invasion and migration [117]. It appears that a reduction in TAM recruitment and the conversion of M2 macrophages to M1 phenotype are effective cancer treatment strategies.

**Figure 5:**
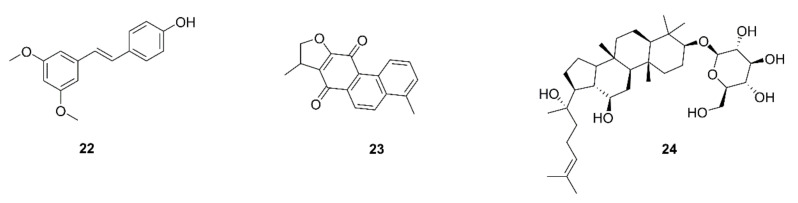
Chemical structures of natural compounds targeting TAMs (**22**–**24**).

MUC1, a kind of pro-oncogenic mucin, is overexpressed in different cancer types and is an indicator of a poorer prognosis [118]. Cancer stem cells (CSCs), key drivers of tumor progression, have the same properties as normal stem cells, such as their self-renewal ability and the potential to transition epithelial into mesenchymal cells [119]. Huang et al. examined the role of MUC1 in TAMs and its connection with the generation of lung CSCs. Significantly increased MUC1 transcription was identified in the lung tissues of lung adenocarcinoma patients compared to those with normal lung tissues. In an experiment involving the coculture of CSCs and M2-TAMs, MUC1 and cancer stemness genes were shown to significantly increase. Pterostilbene (**22**), a polyphenol isolated from the heartwood of red sandalwood (*Pterocarpus santalinus*), is an analog of resveratrol. Huang et al. provided evidence that pterostilbene suppresses M2-polarization through MUC1 inhibition and reduces M2-TAM-induced CSC generation [64]. A study found that dihydroisotanshinone I (DT, **23**), an active ingredient of *Salvia miltiorrhiza*, improves the survival rate of advanced lung cancer patients. This research also indicated that DT has the capability to suppress the migration of A549 and H460 cells, cells cultured with the macrophage medium, and lung cancer/macrophage coculture. DT was shown to inhibit the macrophage recruitment of lung cancer cells by decreasing the expression of chemokine (C-C motif) ligand 2 (CCL2) secreted from both lung cancer cells and macrophages. CCL2 has been recognized as the strongest chemoattractant involved in macrophage recruitment and is a powerful initiator of inflammation [120]. Notably, the CCL2 signaling pathway is a prominent mechanism through which TAMs promote the growth and metastasis of lung cancer cells through bidirectional interactions between lung cancer cells and macrophages [121]. DT was shown to interrupt the crosstalk between macrophages and lung cancer cells [65]. It may be an attractive strategy for transforming TAMs from surface M2 to M1 in the tumor microenvironment [122]. Ginsenoside Rh2 (**24**), an active component of ginseng, has been proven to have the potential to convert M2 macrophages into the M1 subset. A549 and H1299 cells were induced to secrete and express high levels of VEGF-C, MMP-2, and MMP-9 following coculture with M2 macrophages derived from RAW264.7 cells in vitro. In contrast, treatment with ginsenoside Rh2 decreased the secretion and expression of VEGF-C, MMP-2 and MMP-9 and inhibited the proliferation and migration of lung cancer cells. A flow cytometry assay showed a decrease in the M2 phenotype marker CD206 but an increase in the M1 macrophage marker CD16/32 following ginsenoside Rh2 treatment. Furthermore, in C57BL/6 mice subcutaneously injected LLC, ginsenoside Rh2 treatment reduced the expression of the VEGF-C and M2 macrophage marker CD206 [66]. Chemical structures of compounds targeting TAMs are displayed in Figure 5.

The sea hare is a marine organism with various active secondary metabolites [123]. Sea fare hydrolysate was shown to induce the polarization of M1 macrophages and decrease M2 polarization in both RAW264.7 cells and mouse peritoneal macrophages. Sea fare hydrolysate was found to upregulate M1 markers (IL-1β, IL-6, and TNF-α) and downregulate M2 markers (CD206, CD209 and FN-1) in human macrophages and TAMs. In addition, sea fare hydrolysate was shown to inhibit A549 cell growth when cocultured with either M1 cells or M2 cells. Different from most natural products, sea fare hydrolysate was shown to induce M1 and M2 polarization in macrophages, not only in one direction. It might be an effective cancer therapy [67].

Two traditional Chinese medicines were used to treat lung disease in ancient China. Yu-Ping-Feng (YPF), consisting of *Astragalus membranaceus*, *Atractylodes macrocephala,* and *Saposhnikovia divaricate*, has been shown to prolong the survival of orthotopic LLC mice. The percentages of M1 macrophages and CD4^+^ T cells in spleen and tumor tissues were found to increase following YPF administration. YPF was also shown to enhance the cytotoxicity of CD4^+^ T cells and macrophage-mediated LLC lysis [68]. Another Chinese medicine formula, Bu-Fei-Decoction (BFD) was shown to dose-dependently inhibit the ability of A549 and H1975 cells, which were increased by exposure to a conditioned medium from TAMs, to undergo proliferation, migration, and invasion. PD-L1 expression was found to be promoted by IL-10 secreted from TAMs. BFD was shown to decrease the expression of CD206, PD-L1, and IL-10 in lung cancer cells. Thus, BFD has been shown to block crosstalk between TAMs and cancer cells by inhibiting the expression of PD-L1 and IL-10 in vivo and in vitro [69].

### 2.5. Natural Products as Important Immune Checkpoint Inhibitors

The immune checkpoint pathway is a series of cell–cell interactions that function to inhibit the hyperactive effector T cells under normal conditions and prevent attacks on normal cells. However, cancer cells can escape immune destruction by dysregulating the immune response [124]. The most frequently studied immune checkpoint molecules related to lung cancer are PD-1 (programmed cell death protein 1) and CTLA4 [125]. Immune checkpoint inhibitors are monoclonal antibodies developed for corresponding immune checkpoints. Their main function is to block interactions between tumor cells expressing immune checkpoints and immune cells to block the inhibitory effect of tumor cells on immune cells [126,127]. The development of checkpoint-blockade-based immunotherapies has provided more options and attractive weapons in the battle against cancer [128]. However, the use of immunotherapeutic drugs for the treatment of lung cancer can lead to immune-mediated toxicity conditions such as pneumonia, endocrine disease, nephritis, colitis, and pulmonary toxicity [2,129]. Most natural products have low toxicity and high effectiveness. Natural small active molecules have better permeability than monoclonal antibody-based drugs. Due to their multi-component and multi-target characteristics, plant preparations can be combined with monoclonal antibody targeted drugs to achieve an increased efficiency and reduced toxicity.

Many scholars have reported that various natural products have inhibitory effects on programmed cell death ligand 1 (PD-L1) within the TME. Rawangkan et al. reported that green tea extract reduces the percentage of PD-L1 positive cells in lung tumor tissues and the average number of tumors per mouse treated with 4-(methylnitrosamino)-1-(3-pyridyl)-1-butanone, a tobacco-specific carcinogen. In an in vitro experiment, EGCG and green tea extract were shown to downregulate IFN-γ-induced PD-L1 protein expression through JAK2/STAT1 and EGFR/Akt pathways in A549 cells. They were also shown to inhibit EGF-induced PD-L1 expression in Lu99 cells. In a coculture experiment, EGCG was shown to reduce the mRNA expression of PD-L1 in F10-OVA cells and partially restore the mRNA expression of IL-2 in tumor specific T cells [70]. IL-2 is considered as a key molecule in the promotion of T cell proliferation and differentiation, so it has also been called T cell growth factor for decades [130]. Unexpected and interestingly, a recent study showed that IL-2 regulates tumor-reactive CD8^+^ T cell exhaustion in the middle and late tumor stages [131]. Another widely studied active natural compound is berberine (**25**), which is selectively bound to glutamate and inhibits the PD-1/PD-L1 axis through its deubiquitination effect, leading to the ubiquitination and degradation of PD-L1 [71]. Existing evidence shows that the activation of NF-κB promotes the proliferation of regulatory T cells (Treg), leading to the transcription of PD-L1 by binding to its promoter [132]. Ginsenoside Rk1 (**26**), a bioactive ingredient of ginseng, has been shown to downregulate the protein expression of PD-L1 by targeting the NF-κB signaling pathway in A549 and PC9 cells as well as in an A549-xenograft nude mouse model. In addition, ginsenoside Rk1 was shown to induce apoptosis and cell cycle arrest in lung cancer cells [72]. Platycodin D (**27**), a triterpenoid saponin isolated from the southeast Asian functional food *Platycodon grandifloras*, was reported to trigger the extracellular release of PD-L1, leading to a reduction in the inhibition of immunity [73]. However, exosomes carrying PD-L1 in the tumor microenvironment secreted by tumor cells were shown to transfer to distant places where they exerted immunosuppression effects [133]. The targeted inhibition of PD-L1 is particularly important.

**Figure 6:**
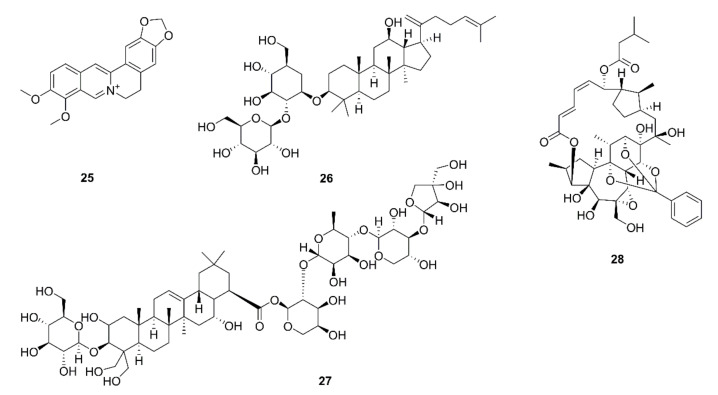
Chemical structures of natural compounds targeting immune checkpoints (**25**–**28**).

A newly discovered human cancer immune checkpoint, CD155, is a cell surface adhesion molecule. The T cell immunoreceptor with Ig and ITIM domains (TIGIT) is an inhibitory receptor that is mainly expressed on NK, Treg, CD4^+^, and CD8+ T cells. The presence of CD155 on the tumor surface combined with TIGIT was shown to inhibit the function of NK and other immune cells [134]. A small molecule, rediocide A (**28**), isolated from *Trigonostemon rediocides*, was shown to reduce the expression of CD155 in A549 and H1299 cells by 11% and 14%, respectively, thus blocking the tumor immune resistance to NK cells [74]. Chemical structures of compounds that exert immune checkpoint inhibitor effects are displayed in Figure 6.

## 3. Combination of Natural Products and Anticancer Drugs

Tumor drug resistance is an obstacle to tumor treatment that may lead to tumor recurrence or treatment failure. More and more evidence is supporting the idea that the combination of natural products and anticancer drugs can have better therapeutic benefits. Natural products have increased efficiency, reduced toxicity, and can induce improved immunity by regulating various signal pathways.

Compared with anlotinib monotherapy, the combination of anlotinib and the traditional Chinese medicine *Brucea Javanica* oil was shown to inhibit the growth and angiogenesis of SCLC liver metastases more significantly. *Brucea Javanica* oil also reduced weight loss in model and normal mice following anotinib treatment [75]. *Mahonia aquifolium* extract was shown to promote the antitumor effects of doxorubicin. The extract was shown to prolong the action time of doxorubicin in A549 cells. The combined application of doxorubicin/extract was shown to decrease MMP-9 expression. A549 cells treated with the extract and doxorubicin combination displayed lower colony and migratory formation potential than untreated cells or cells only treated with doxorubicin. The application of this combination was found to reduce the dosage of doxorubicin required, thereby reducing the toxicity to normal tissues [76]. The combination of Fei-Liu-Ping ointment and cyclophosphamide was shown to suppress lung cancer growth and invasion by inhibiting the tumor inflammatory environment. This suggests that Fei-Liu-Ping ointment can be used alone or in combination with the routine treatment of inflammation-related pneumonia [77]. Liu et al. also proved that the combination of Fei-Liu-Ping ointment with celecoxib inhibits the tumor inflammatory microenvironment in an LLC xenograft model [78]. Disintegrin and a metalloproteinase (ADAM9), a type I transmembrane protein, are overexpressed in various cancers, including lung cancer [135,136,137]. Lin et al. proved that a secreted form of ADAM9 promotes cancer invasion through tumor-stromal interactions [137]. Subsequently, they indicated that resveratrol inhibits the protein expression of ADAM9 in A549 and Bm7 cells through the ubiquitin–proteasome pathway. A synergistic anticancer effect was shown when resveratrol was used in combination with dasatinib or 5-fluorouridine [79]. Studies have shown that the application of carnosic acid, ginsenoside Rh2, and water extract of ginseng enhances the antitumor effects of cisplatin by decreasing PD-L1 expression, inhibiting MDSCs, or regulating macrophage polarization [80,81,82].

## 4. Combination of Natural Products with Nanotechnology or Other Materials for Targeting the TME

Technological advances have led to the development of innovative drug delivery systems [138,139]. Various natural and synthetic materials have been used as potential biomaterial carriers of therapeutic agents in cancer therapy. Tumor treatment requires the localization of active substances in tumor cells. The combination of drugs and materials aids in achieving better localization of active substances and minimizes the impact of active substances on normal cells or maximizes the impact on tumor cells. Some materials themselves have anti-tumor effects, and their combination with drugs enhances the effects of the drugs. Nano-, micro-, and macroscale drug delivery systems are used to improve the bioavailability of drugs [140]. In addition, the combination of polysaccharides with more polymers to improve their required functional properties, such as encapsulation, stability, and release of drugs, is a common practice. Pectin is a natural excellent macromolecule polymer with biocompatible and biodegradable properties that is used for targeted drug delivery [141,142]. Moreover, some experiments have shown that pectin has the ability to inhibit tumor growth in cancers [143,144]. Poly (vinyl pyrrolidone) (PVP) is used in the development of biomedical applications as a complexing agent or cross-linker with excellent biocompatibility and solubility characteristics. Gaikwad et al. prepared pectin-PVP based curcumin particulates of different ratios and evaluated their localized delivery to lung cancer tumors. The results showed the optimal ratio of particles and indicated that it could be used for inhalation in lung cancer treatment. Spray-dried pectin-PVP curcumin was shown to enhance curcumin solubility. It was also shown to inhibit cancer cell proliferation and angiogenesis more than curcumin treatment alone [145]. Singh et al. reported that nanoparticles encapsulating polyphenols, EGCG and theaflavin and combined with cisplatin exhibited more biological effectiveness and stronger inhibition of cell proliferation, metastasis, and angiogenesis biomarkers than EGCG/theaflavin alone.

## 5. Conclusions

Natural products are important sources of new drugs. In this review, we focused on natural products that have been reported to have anticancer activity targeting the TME in lung cancer. Our findings are of great significance for the development of new plant-derived chemotherapy agents for the treatment of lung cancer. Most studies in this area have been related to angiogenesis, MDSCs, and TAMs, and research on some other aspects is lacking. The use of appropriate phytochemicals, medicinal plants, or other natural substances in combination with immune checkpoint inhibitors for lung cancer treatment may be a better choice than using monotherapies. However, there are few reports on combined use in the literature. Moreover, the activity of some natural products is not very high due to problems with their stability and bioavailability. This can be solved by structural modifications or by combining these compounds with material technologies such as nanotechnologies. Moreover, nanoparticle-mediated delivery of natural compounds may limit the unwanted toxicity of chemotherapeutic agents. Unfortunately, few studies have been done on the effects of natural products in combination with other materials on the TME in lung cancer. In addition, the determination of the components of botanical agents and traditional Chinese medicine extracts has been a problem requiring resolution for a long time. Intensified technology is needed to identify natural products and active derivatives and to research potential antitumor effects.

## Figures and Tables

**Table 1:** The effects of natural products on modulation of the TME.

No.	Natural Products	Common Source	Cell Lines or Animal Models or Patients	Function or Molecular Mechanism	Ref.
*Targeting angiogenesis*					
1	Jolkinolide A (**1**)	*Euphorbia* *fischeriana*	A549, HUVEC; A549 cell xenograft mice	Inhibition of the Akt-STAT3-mTOR signaling pathway and reduction of VEGF protein expression; inhibition of HUVEC migration	[10]
2	Jolkinolide B (**2**)				
3	Parthenolide (**3**)	*Tanacetum* *parthenium*	A549, H526	Inhibition of A549 and H526 cell proliferation in the presence and absence of nicotine; induction of apoptosis; inhibition of angiogenesis; down-regulation of Bcl-2 expression and up-regulation of E2F1, p53, GADD45, Bax, Bim, and caspase 3,7,8,9 expression	[11]
4	Galbanic acid (**4**)	*Ferula* *assafoetida*	LLC, HUVEC; LLC-bearing mice	Inhibition of VEGF-inducible HUVECs and LLC proliferation; tube formation, migration/invasion inhibition in HUVECs; decreased phosphorylation of p38MAPK, JNK and Akt, and decreased expression of VEGFR targeting eNOS; inhibition of tumor-induced angiogenesis and tumor growth in mice; reduction of CD34 and Ki67	[12]
5	Salvicine (**5**)	*Salvia* *prionitis*	A549, HMEC	Inhibition of A549 cell viability; inhibition of the migration and tube formation of HMECs; reduced mRNA expression levels of bFGF; VEGF mRNA expression unchanged	[13]
6	Tanshinone IIA (**6**)	*Salvia* *miltiorrhiza*	A549	Proliferation inhibition; apoptosis induction; cell cycle arrest at the S stage; downregulation of protein expression of VEGF and VEGFR2	[14]
7	β-hydroxyisovalerylshikonin (**7**)	*Lithospermum* *erythrorhizon*	HUVEC, VPC; LLC xenograft mice	Inhibition of blood vessel formation by chicken chorioallantoic membrane assay; suppression of in vivo angiogenesis; suppression of VEGFR2 and Tie2; inhibition of HUVECs and VPC growth; suppression of MAPK and Sp1-dependent VEGFR2 and Tie2 mRNA expression	[15]
8	Isogambogenic acid (**8**)	*Gamboge* *hanburyi*	A549, HUVEC; transgenic FLK-1 promoter EGFP zebrafish; A549 xenograft mice	More effective for inhibiting HUVEC proliferation than A549; angiogenesis inhibition in zebrafish embryos; suppression of angiogenesis and tumor growth in mice; inhibition of vessel sprouting ex vivo; inhibition of VEGF-induced migration, invasion, and tube formation, morphological changes in HUVECs	[16]
9	Tubeimoside-1 (**9**)	*Bolbostemma paniculatum*	H460, A549, eEND2; H460 xenograft mice	Inhibition of tumor growth and vascularization; vascular sprouting; eEND2, H460, and A549 cell viability; eEND2 cell migration; VEGFR2 and Tie2 expression; and the Akt/mTOR pathway	[17]
10	Decursin (**10**)	*Angelica gigas*	HUVEC, LLC; LLC xenograft mice	Inhibition of VEGF-induced HUVEC proliferation, angiogenesis, and blood vessel formation; suppression of VEGF-induced phosphorylation of p42/44 ERK and JNK MAPK in endothelial cells and VEGF-induced MMP-2 activation; suppression of tumor growth and angiogenesis in nude mice	[18]
11	Decursinol angelate (**11**)				
12	Farnesiferol C (**12**)	*Ferula* *assafoetida*	HUVEC, LLC; LLC allograft tumor mice	Inhibition of VEGF-induced proliferation, migration, invasion, and MMP-2 secretion of HUVECs; inhibition of VEGF-induced vessel sprouting ex vivo; inhibition of tumor growth, angiogenesis, proliferation in vivo; inhibition of VEGF-induced phosphorylation of p125 FAK (pY861), Src (pY416), ERK1/2, p38MAPK, and JNK	[19]
13	Ergosterol (**13**)	*Agaricus blazei*	s180, LLC; s180 and LLC-bearing mice; mice inoculated with matrigel	Prevention of neovascularization induced by both LLC cells and matrigel; inhibition of tumor growth	[20]
14	Grape seed proanthocyanidins (GSP)		A549, H1299, H226, H460, H157, H1975, H1650, H3255, HCC827, BEAS-2B; A549 and H1299 xenograft mice	Inhibition of the proliferation of human lung cancer cells but not normal human bronchial epithelial cells; GSP-induced inhibition of proliferation is blocked by IGFBP-3 knockdown; inhibition of tumor growth and neovascularization; upregulation of IGFBP-3 protein levels in plasma and lung tumors; inhibition of VEGF expression	[21,22,23,24,25]
15	Pomegranate fruit extract		B(a)P-induced lung tumorigenesis in A/J mouse	Inhibition of the activation of NF-κB, IKKα, PI3k and mTOR, and phosphorylation of IκBα, MAPKs, Akt and c-met; down-regulation of Ki-67, PCNA, CD31, VEGF, and iNOS expression	[26]
16	Extracts of *Astragali Mongolici* and *Rhizoma Curcumae*		LLC-bearing mice	Decreases in tumor weight and tumor MVD; down-regulation of p38 MAPK, p-p38 MAPK, ERK1/2, p-ERK1/2, JNK, p-JNK, and VEGF expression	[27]
17	*Scutellaria barbata* extract		CL1-5, HEL299, 293T, LL2, HMEC-1; LL2 lung metastatic mice	Decrease in the transcriptional activity of HIF-1α by inactivation of AKT; inhibition of VEGF expression in CL1-5 cells, migration and proliferation of HMEC-1 cells; inhibition of tumor growth	[28]
18	*Ginkgo biloba* exocarp extracts		LLC; LLC transplanted tumor mice	Inhibition of LLC cell proliferation; downregulation of CD34, Wnt3α, β-catenin, p-AKT/AKT, VEGF, and VEGFR2 expression	[29]
19	Green tea extract		NNK-induced lung tumorigenesis in mouse	Decreases in CD31, MVD and VEGF expression; apoptosis	[30]
20	An-te-xiao capsule	Chinese medicine	A549, H460, H520, LLC, HUVEC; LLC xenograft mouse; H460 and H520 xenograft mice	No acute oral toxicity; prolongation of survival time; inhibition of tumor growth; decreases in MVD, CD31, and the blood vessel number; inhibition of Td-EC migration, invasion, and tube formation in the presence or absence of VEGF; inhibition of VEGF secretion and VEGFR2 phosphorylation	[31]
21	Erbanxiao solution	Chinese medicine	Patients with lung cancer	Inhibition of tumor angiogenesis by changing the levels of VEGF, bFGF, and TNF-α	[32]
22	Ka-mi-kae-kyuk-tang	Korean herbal cocktail	HUVEC, LLC; LLC-bearing mice; PC-3 xenograft mice	Suppression of bFGF stimulated endothelial membrane receptor-tyrosine kinase signaling to ERK1/2, cell motility, capillary differentiation, and to a less extent mitogenesis; inhibition of HIF1α and VEGF; suppression of tumor growth;	[33]
23	Qingzaojiufei decoction	Chinese medicine	LLC	Inhibition of LLC proliferation and growth; up-regulation of p53, and down-regulation of c-myc and Bcl-2; reducion of MMP-9, VEGF, VEGFR, p-ERK1/2	[34]
24	Yiqichutan formula	Chinese medicine	A549, H460, H446; A549-bearing rats, H460-bearing rats, H446-bearing rats	Inhibition of tumor growth; reductions in CD31 expression and the number of blood vessels; decreases in VEGF, HIF-1, DLL4, and Notch-1 protein expression and VEGF mRNA expression	[35]
*Targeting the ECM*					
25	Curcumin (**14**)	*Curcuma logna*	A549; A549 xenograft mice	Attenuation of the GLUT1/MT1-MMP/MMP2 pathway	[36]
26	Honokiol (**15**)	*Magnolia* *officinalis*	H1299	Disruption of HDAC6-mediated Hsp90/MMP-9 interaction; MMP-9 protein degradation; inhibition of migration, invasion, and MMP-9 proteolytic activity and expression; regulation of ubiquitin proteasome system	[37]
27	Theaflavin (**16**)	Black tea	LL2-Lu3	Inhibition of cell invasion, MMP-2 and MMP-9 secretion, and type IV collagenases	[38]
28	Theaflavin digallate (**17**)				
29	EGCG (**18**)	Green tea	CL1-5, CL1-0	Cell cycle G2/M arrest; inhibition of cell invasion and migration; repression of MMP-2 and -9 activities; reduction of nuclear translocation of NF-κB and Sp1; JNK signaling pathway	[39,40]
			A549, HUVEC; A549 xenograft mice	Inhibition of nicotine-induced migration and invasion; down-regulation of HIF-1α, VEGF, COX-2, p-Akt, p-ERK, and vimentin protein levels; up-regulation of p53 and β-catenin protein levels; suppression of HIF-1α and VEGF protein expression	[41]
30	Steroidal saponins extracted from *Paris polyphylla*		A549	Suppression of cell proliferation, adhesion, migration, and invasion; downregulation of MMP-2 and -9 protein levels; inhibition of MMP-2 and -9 activity	[42]
31	Methanolic extract of *Euchelus asper*		A549; chick chorio-allantoic membrane model	Cell cycle subG1 phase arrest; reduction of A549 proliferation, MMP-2 and -9; decrease of the branching points of the 1st order blood vessels or capillaries of the chorio-allantoic membrane	[43]
32	*Phyllanthus urinaria* extract		A549, LLC; LLC-bearing mice	Cytotoxicity; inhibition of invasion and migration; inhibition of u-PA, and MMP-2 and -9 activity; inhibition of TIMP-2 and PAI-1 protein expression; inhibition of transcriptional activity of MMP-2 promoter; inhibition of p-Akt, NF-κB, c-Jun, and c-Fos; decrease in lung metastases	[44]
33	*Rosa gallica* petal extract		A549	Downregulation of the PCNA, cyclin D1, and c-myc; suppression of cell migration and invasion; inhibition of the expression and activity of MMP-2 and -9; regulation of EGFR-MAPK and mTOR-Akt signaling pathways	[45]
34	*Viola Yedoensis* extract		A549, LLC	Cytotoxicity; inhibition of cell invasiveness and migration; suppression of MMP-2, -9 and u-PA; decreases in TIMP-2 and TIMP-1 protein levels; increase in PAI-1 protein expression; decreasein NF-κB DNA binding activity	[46]
35	*Cinnamomum cassia* extract		A549, H1299	Cytotoxicity; inhibition of cell migration, invasiveness and motility; inhibition of MMP-2 u-PA, and RhoA protein levels; decrease of cell-matrix adhesion to gelatin and collagen; decrease of FAK and ERK1/2 phosphorylation;	[47]
36	*Duchesnea indica* extracts		A549, H1299	Inhibition of MMP-2 and u-PA activity; cell invasion and metastasis inhibition; downregulation of the expression of p-ERK, p-FAK Tyr397, p-paxillin Tyr118, c-Jun, c-Fos, and TGF-b1 induced-vimentin; inhibition of tumor growth	[48]
37	Fructus phyllanthi tannin fraction		H1703, H460, A549, HT1080	Cytotoxicity; inhibition of cell migration and invasion; down-regulation of p-ERK1/2, MMP-2 and -9 expression level, up-regulation of p-JNK expression; regulation of the MAPK pathway	[49]
38	Butanol fraction extract of *Psidium cattleianum* leaf		H1299	Suppression of activities, protein and mRNA expression levels of MMP-2 and -9; inhibition of adhesion, migration and invasion; downregulation of the mRNA level of uPAR; suppression of the ERK1/2 signaling pathway	[50]
39	*Terminalia catappa* leaf extract		A549, LLC	Absence of cytotoxicity; inhibition of invasion, metastasis and motility; inhibition of u-PA, MMP-2 and -9 activity; inhibition of protein levels of TIMP-2 and PAI-1	[51]
40	*Rhizoma Paridis* saponins		LA795 xenograft mice	Inhibition of tumor growth; downregulation of mRNA expression of MMP-2 and -9 and ascendance of TIMP-2	[52]
41	*Selaginella tamariscina* extract		A549, LLC; LLC-bearing mice	Inhibition of invasion and motility; reduced activity of u-PA MMP-2 and -9; increase of protein levels of TIMP-2 and PAI-1 in A549 cells; decrease in lung metastases in mice	[53]
42	Ethanol extract of *Ocimum sanctum*		LLC; LLC lung metastasis mouse model	Cytotoxicity; inhibition of cell adhesion and invasion; inhibition of nodules mediated by LLC cells; decrease in the activity of the enzymes SOD, CAT, and GSH-Px	[54]
43	Rhubarb serum metabolites		A549; A549 lung metastatic mouse model	Suppression of the activity and expression level of MMP-2; inhibition of NF-κB/c-Jun pathway; inhibition of u-PA expression; inhibition of cell motility in vitro and lung metastasis *in vivo*	[55]
44	Fuzheng Kang-Ai decoction	Chinese medicine	A549, PC9, H1650	Suppression of cell proliferation; inhibition of cell migration and invasion; downregulation of MMP-9 activity and protein expression; downregulation of EMT related protein N-cadherin and vimentin	[56]
45	Yifei Tongluo	Chinese medicine	LLC-bearing mice	Inhibition of tumor growth; prolonged survival; fewer nodules on the lung surface; inhibition of MVD, CD34, VEGF, MMP-2, MMP-9, N-cadherin, and vimentin; increases in E-cadherin expression and NK cytotoxic activity; increased percentages of CD4^+^, CD8^+^ T, and NK cells; upregulation of Th1-type cytokines and levels of IFN-γ and IL-2 in the serum and reduction in IL-10 and TGF-β1; downregulation of PI3K/AKT, MAPK, and TGFβ/Smad2 pathways; upregulation of the JNK and p38 pathways	[57]
*Targeting MDSCs*					
46	Resveratrol (**19**)	Grape skin and seeds	LLC; LLC-bearing mice	Decrease in G-MDSC accumulation by triggering its apoptosis and decreasing recruitment; promotion of CD8^+^IFN-γ^+^ cell expansion; impairing the suppressive capability of G-MDSC on CD8^+^ T cells; G-MDSC differentiation into CD11c^+^orF4/80^+^ cells	[58]
47	Silymarin (**20**)	*Silybum marianum*	LLC-bearing mice	Inhibition of tumor growth; apoptosis; increases in the infiltration and function of CD8^+^ T cells; increases in IFN-γ and IL-2 levels, decrease in the IL-10 level	[59]
48	Vitamin D (**21**)	Sea fish, animal liver, etc.	COVID-19 patients	Beneficial effects come from reducing the macrophage and MDSC hyperinflammatory response	[60]
49	Polysaccharide from *Ganoderma lucidum*		LLC-bearing mice	Inhibition of tumor growth; reduction of MDSC accumulation in spleen and tumor tissue; increase in the percentage of CD4^+^, CD8^+^ T cells and IFN-γ and IL-12 production in the spleen; decreases in arginase activity and NO production; increase in IL-12 production in tumor tissue; regulation of the CARD9-NF-κB-IDO pathway in MDSCs	[61]
50	Curdlan produced by *Alcaligenes faecalis*		LLC-bearing mice	Promotion of MDSC differentiation; impairment of the suppressive capability of MDSCs; inhibition of tumor progression by reducing MDSCs and enhancing the CTL and Th1 responses;	[62]
51	Ze-Qi-Tang formula	Chinese medicine	LLC; orthotopic lung cancer mouse model	G-MDSC apoptosis through the STAT3/S100A9/Bcl-2/Caspase-3 signaling pathway; elimination of MDSCs and enrichment of antitumor T cells; inhibition of tumor growth; prolongation of survival	[63]
*Targeting TAMs*					
52	Pterostilbene (**22**)	*Pterocarpus santalinus*	A549, H441	Decrease in the induction of stemness by M2-TAMs; prevention of M2-TAM polarization and decrease in side-population cells; suppression of the self-renewal ability in M2-TAMs-co-cultured lung cancer cells accompanied by down-regulation of MUC1, NF-κB, CD133, β-catenin, and Sox2 expression	[64]
53	Dihydroisotanshinone I (**23**)	*Salvia miltiorrhiza*	A549, H460	Inhibition of cell motility and migration; blockage of the macrophage recruitment ability of lung cancer cells; inhibition of CCL2 secretion; blockage of p-STAT3	[65]
54	Ginsenoside Rh2 (**24**)	Ginseng	A549, H1299	Inhibition of cell proliferation and migration; decrease in the secretion and mRNA and protein levels of VEGF-C, MMP-2, and -9; decreases in VEGF-C and CD206 expression by tumor tissues	[66]
55	Sea fare hydrolysate		A549, HCC-366, RAW264.7,	Polarization of M1 macrophages in RAW264.7 cells; reduction of IL-4-induced M2 polarization in mouse peritoneal macrophages with reductions in the M2 markers Arg-1 and Ym-1; suppression of M2 macrophage polarization in human TAMs with reductions in the M2 markers CCL18, CD206, CD209, fibronectin-1, and IL-10 and increases in the M1 markers IL-1, IL-6, and TNF-α; reductions in the activity of STAT3 and p38 in TAMs; cytotoxicity and G2/M arrest	[67]
56	Yu-Ping-Feng	Chinese medicine	orthotopic lung tumor-bearing mice	Survival prolongation; increases in the CD4^+^ T Cell and M1 macrophage populations; cytotoxicity of CD4^+^ T cells; enhancement of the Th1 immunity response; STAT1 activation in M1 macrophages	[68]
57	Bu-Fei- Decoction	Chinese medicine	A549, H1975; A549 and H1975 xenograft mice	Suppression of cell proliferation, migration, and invasion in TAM conditioned medium; reduced expression of IL-10 and PD-L1; suppression of A549 and H1975 tumor growth, and PD-L1, IL-10 and CD206 protein expression in xenograft mice	[69]
*Targeting immune checkpoint*					
58	Green tea extract and EGCG		A549, Lu99; NNK-induced lung tumor mice	Downregulation of IFN-γ-induced PD-L1 protein; inhibition of STAT1 and Akt phosphorylation; inhibition of the IFNR/JAK2/STAT1 and EGFR/Akt signaling pathway; reduction of PD-L1-positive cells and inhibition of tumor growth in mice	[70]
59	Berberine (**25**)	*Coptis chinensis*	A549, H157, H358, H460, H1299, H1975, LLC, Jurkat; Lewis tumor xenograft mice	Negative regulator of PD-L1; decrease in PD-L1 expression; recovery of the sensitivity of cancer cells to T-cell killing; suppression of xenograft tumor growth; tumor-infiltrating T-cell activation; PD-L1 destabilization by binding to and inhibition of CSN5 activity; inhibition of CSN5 activity by directly binding to CSN5 at Glu76	[71]
60	Ginsenoside Rk1 (**26**)	Ginseng	A549, PC9; A549 xenograft mice	Inhibition of cell proliferation and tumor growth; cell cycle arrest in the G1 phase; apoptosis via the NF-κB signaling pathway; downregulation of PD-L1 and NF-κB expression	[72]
61	Platycodin D (**27**)	*Platycodon grandifloras*	H1975, H358	Decrease in the PD-L1 protein level; increase in IL-2 secretion; extracellular release of PD-L1 independent of the hemolytic mechanism	[73]
62	Rediocide A (**28**)	*Trigonostemon rediocides*	A549, H1299	Blockage of cell immuno-resistance; increase in granzyme B release and IFN-γ secretion; down-regulation of CD155 expression	[74]

**Table 2:** The effects of natural products combined with chemotherapy drugs on modulation of the TME.

Natural Products	Combined Clinical Drugs	Cell Lines or Animal Models	Function or Molecular Mechanism	Ref.
*Brucea Javanica* oil	Anlotinib	H446; H460 liver-metastasis mouse model	Enhancement of anlotinib efficacy against liver metastasis from SCLC; reduction of anlotinib-induced weight loss in mice; enhancement of the anti-angiogenic effect (inhibition of tumor microvessels growth) of amlotinib	[75]
*Mahonia aquifolium* extract	Doxorubicin	A549	Increased cytotoxicity; cell cycle arrest in the subG1 phase; pronounced DOX retention; lower migratory ability and colony formation potential; decrease in MMP-9 expression	[76]
Fei-Liu-Ping ointment	Cyclophosphamide	A549, THP-1; LLC xenograft mice	Enhancement of tumor growth inhibition; down-regulation of the inflammatory cytokines TNF-α, IL-6 and IL-1β levels; increase in E-cadherin expression and decrease in N-cadherin and MMP-9, expression; inhibition of cell proliferation and invasion; inhibition of NF-κB activity, expression, and nuclear translocation	[77]
	Celecoxib	LL/2-luc-M38; LLC xenograft mice	Enhancement of tumor growth inhibition; inhibition of Cox-2, mPGES-1, VEGF, PDGFRβ, MMP-2, and -9 expression; down-regulation of E-cadherin expression, upregulation of N-cadherin and Vimentin expression	[78]
Resveratrol	Dasatinib, 5-fluorouridine	A549, Bm7	Inhibition of cell migration; ADAM9 degradation via the ubiquitin-proteasome pathway; synergistic anticancer effects to inhibit cell proliferation	[79]
Carnosic acid	Cisplatin	LLC-bearing mice	Enhancement of tumor growth suppression and apoptosis; reduction of side effects (body weight loss) of cisplatin; promotion of CD8^+^ T cells-mediated antitumor immune response; function and accumulation decrease of MDSCs; downregulation of CD11b^+^ Gr1^+^ MDSCs, Arg-1, iNOS-2, and MMP-9 levels	[80]
Ginsenoside Rh2	Cisplatin	A549, H1299	Enhancement of cisplatin-induced cell apoptosis by repressing autophagy; scavenging of cisplatin-induced superoxide autophagy generation; inhibition of cisplatin-induced EGFR-PI3K-AKT pathway activation; inhibition of the cisplatin-induced PD-L1 expression	[81]
Water extract of ginseng	Cisplatin	A549, THP-1; LLC-bearing mice	Increase in the expression of the M1 macrophage marker iNOS, decrease in the expression of the M2 marker Arg-1; regulation of TAMs polarization; reductions in tumor growth and cisplatin-induced immunosuppression	[82]
